# Translational perspectives on neurophysiological responses and mechanisms in blood flow restriction exercise

**DOI:** 10.1113/EP093587

**Published:** 2026-06-16

**Authors:** Kai T. Fox, Luke Hughes, Brendan R. Scott, Paul S. R. Goods, Shaun YM. Teo, Ann‐Maree Vallence

**Affiliations:** ^1^ Centre for Healthy Ageing Health Futures Institute Murdoch University Perth Australia; ^2^ Physical Activity, Sport and Exercise (PHASE) Research Group, School of Allied Health (Exercise Science) Murdoch University Perth Australia; ^3^ Aerospace Medicine and Rehabilitation Laboratory, School of Sport, Exercise, and Rehabilitation Northumbria University Newcastle upon Tyne UK; ^4^ School of Psychology, College of Health and Education Murdoch University Perth Australia

**Keywords:** functional ability, hypoxia, ischaemia, neuroplasticity

## Abstract

Maintaining corticospinal tract function is crucial for voluntary movement. Exercise using high external loads/intensities is a promising intervention to promote adaptations to the corticospinal tract and improve physical function; however, such loads/intensities may not be tolerable or safe in populations with neurological disorders, motor‐impaired injuries and older adults. Exercise using low external loads/intensities with blood flow restriction offers an alternative that provides comparable adaptations in muscular hypertrophy, strength and function (e.g., gait performance) to higher external‐load/intensity exercise. Although some data suggest that blood flow restriction exercise may induce neuroplasticity, the neurophysiological responses remain largely unknown. Given the potential significance of blood flow restriction exercise for clinical populations, this review synthesises available data and potential mechanisms of these neuroplastic responses while providing translational insights and future research recommendations.

## Abstract (highlights)

- **What is the topic of this review?**
  This short communication synthesises evidence on the neurophysiological (specifically neuroplastic) responses to blood flow restriction exercise, the potential mechanisms of these responses and their functional relevance for neurorehabilitation.
- **What advances does it highlight?**
  Blood flow restriction exercise may promote functionally beneficial neuroplasticity, likely attributed to the heightened metabolic stimuli within the distal musculature that enhance group III afferent firing during active contraction and sustain group IV afferent firing throughout the protocol. Future studies are required to comprehensively assess facilitatory and inhibitory circuits both cortically and subcortically, with a priority on chronic neurophysiological adaptations.

## 1 INTRODUCTION

The corticospinal tract (CST) is the primary pathway controlling voluntary movement, comprising neuronal axons within the primary motor cortex that transmit descending volleys to spinal circuits, including α‐motoneurons innervating skeletal muscle (Javed et al., 2018; Lemon, 2019). Maintaining CST function is critical for voluntary movement and functional independence. Impairments in CST and motor function occur with physical inactivity (Hassanlouei et al., 2017), ageing (Clark & Taylor, 2011) and various pathologies, including spinal cord injuries (Benedetti et al., 2022) and neurological conditions (Badawy et al., 2013). Neuroplasticity, the nervous system's ability to reorganise its structure and function in response to environmental and physiological stimuli, is important for improving CST function (Cramer et al., 2011). Inasmuch as ∼1.8 billion people are physically inactive (World Health Organisation, 2024b), ageing is inevitable, and over 3 billion people are affected by neurological conditions worldwide (World Health Organisation, 2024a), rehabilitation tools to induce neuroplasticity and improve CST function are essential.

Exercise using high external loads/intensities elicits adaptations in corticospinal excitability, intracortical inhibition and spinal excitability, alongside improved functional ability (Gomez‐Guerrero et al., 2024; Siddique et al., 2020). However, such exercise is not always tolerable or safe within motor‐impaired populations, presenting a barrier to exercise‐induced neuroplasticity. An alternative is low external‐load/intensity exercise with blood flow restriction (BFR), a safe training technique when appropriate application and pre‐exercise screening are completed (see Patterson et al., 2019). During BFR, pneumatic tourniquet cuffs are placed proximally on the exercising limbs and inflated to a pre‐determined pressure to compress the limb and partially restrict arterial inflow whilst restricting venous outflow, inducing localised hypoxia in distal musculature and other tissues (Scott et al., 2015). Pressures should be individualised to the minimum pressure required to completely restrict arterial inflow into the limb, that is, ‘limb occlusion pressure’ (LOP) measured using the tourniquet cuff as a sensor (Hughes & McEwen, 2021) or Doppler ultrasound of an artery (arterial occlusion pressure: AOP) (Frechette et al., 2025). The hypoxic environment increases reliance on anaerobic metabolism, increasing metabolite accumulation (e.g., inorganic phosphate), with the restricted venous outflow slowing metabolite removal (Suga et al., 2012), facilitating physiological responses, such as increased type II motor unit recruitment, and training adaptations (Pearson & Hussain, 2015). Recent data suggest that low external‐load/intensity resistance or aerobic exercise may induce functionally beneficial neurophysiological adaptations, including increased neural drive (Centner & Lauber, 2020), increased corticospinal excitability (Brandner, Warmington et al., 2015; Frechette et al., 2025) and reduced cortical inhibition (Norbury et al., 2024). This short communication synthesises current evidence on neurophysiological responses to BFR exercise and the functional implications that may warrant its use within neurorehabilitation, specifically in individuals who may not be able to tolerate near‐maximal exertion, for instance, older adults, or those with a musculoskeletal injury or neurological disorder.

## 2 AFFECTED MECHANISMS OF BFR EXERCISE

Increased accumulation of inorganic phosphate and lactate, and decreased pH, during BFR exercise represent primary physiological responses for subsequent training adaptations. These metabolic perturbations facilitate secondary mechanisms contributing to chronic adaptations; namely, systemic hormonal responses, cell swelling, anabolic/anti‐catabolic signalling and enhanced type II fibre recruitment (Pearson & Hussain, 2015). However, the processes underlying potential neurophysiological adaptations remain poorly understood.

The enhanced recruitment of higher‐threshold motor units and increased firing rates during BFR exercise (Fatela et al., 2019) may be attributed to enhanced afferent feedback. Group IV afferents are metabosensitive (i.e., sensitive to intramuscular metabolites) and group III afferents are primarily mechanosensitive, though metabolic stimuli heighten their responsiveness (Hayes et al., 2006). Given that afferents influence spinal and cortical structures (Copithorne et al., 2020; Latella et al., 2020, 2021), metabolite accumulation during BFR exercise may enhance group IV afferent activation and increase group III afferent firing during contractions, facilitating or inhibiting spinal and/or supraspinal motoneurons via ascending pathways. Although group III/IV afferents cannot be directly assessed in vivo, indirect evidence supports this suggestion. Notably, studies using complete BFR (i.e., full occlusion) following fatiguing exercise describe sustained group III/IV afferent firing (Copithorne et al., 2020; Latella et al., 2020, 2021). However, animal model evidence suggests metabolic stimuli primarily enhance group III afferent activation during contraction; therefore, speculatively, post‐exercise BFR primarily maintains group IV afferent firing (Hayes et al., 2006). Studies have used complete BFR following fatiguing exercise to activate afferent feedback and examined its influence on corticospinal excitability using transcranial magnetic stimulation (TMS) (Latella et al., 2020, 2021). TMS is a non‐invasive brain stimulation technique measuring corticospinal excitability via motor evoked potential (MEP) amplitude, and excitability of intracortical facilitatory and inhibitory circuits in the primary motor cortex (Hallett, 2007). For instance, MEP amplitude decreased following fatiguing quadricep exercise without BFR, but remained unchanged during post‐exercise BFR (∼250 mmHg), indicating that afferent feedback during BFR influences the descending CST drive in the presence of central fatigue (Latella et al., 2021). Following fatiguing upper limb contractions, intracortical facilitation was reduced without restriction but preserved during complete post‐exercise BFR (Latella et al., 2020), suggesting that afferent feedback during BFR attenuates fatigue‐related declines in intracortical facilitatory circuits by activating glutamate excitatory neurotransmitters within the primary motor cortex (Liepert et al., 1997). These supraspinal responses may explain region‐specific (primary and premotor cortex) increases in cortical oxygenation, via functional near‐infrared spectroscopy, as a proxy for cortical activation during BFR squatting (Jia et al., 2024).

Some data suggest that sustained afferent firing may preferentially influence spinal structures. Despite no differences in MEPs normalised to spinal excitability between unrestricted and BFR conditions, Copithorne et al. (2020) reported significantly greater increases in spinal motoneuronal excitability at the bicep brachii immediately following fatiguing exercise with BFR (250 mmHg) compared to the unrestricted fatiguing protocol (∼147% difference). Interestingly, post‐exercise BFR (300 mmHg) reduced spinal excitability projecting to the triceps brachii (∼20%) following elbow flexion fatigue, whereas spinal excitability of the biceps brachii was increased (∼25%) following elbow extension fatigue, with differences attributed to differential properties of the α‐motoneuron pools projecting to extensor or flexor muscles over differences in afferent feedback (Martin et al., 2006). Although corticospinal responses are heterogeneous, the data suggest that BFR can modulate CST activity, likely via enhanced group III afferent firing during contractions and sustained group IV afferent firing due to irremovable metabolic stimuli.

## 3 NEUROPHYSIOLOGICAL RESPONSES TO BFR EXERCISE

Early literature theorised that neuromuscular adaptations to BFR resistance training occur later than those from traditional high external‐load resistance exercise (BFR, >9 weeks vs. traditional resistance training, <6 weeks) (Loenneke et al., 2012). However, a meta‐analysis reported larger increases in neural drive following ≥4 weeks of low external‐load BFR resistance training compared to unrestricted low external‐load training (Centner & Lauber, 2020). Additionally, BFR resistance training elicited comparable increases in neural drive to high external‐load training following programmes exceeding 6 weeks. These findings suggest that BFR exercise increases neural drive through adaptations along descending corticospinal pathways on a similar timescale to high external‐load exercise.

Acute research offers mechanistic insight into chronic increases in neural drive following BFR training. Acute studies show increased corticospinal excitability (Brandner, Warmington et al., 2015; Frechette et al., 2025) and reduced inhibition (Norbury et al., 2024), potentially reflecting increases in synaptic strength (i.e., long‐term potentiation), though one study refutes any corticospinal responses following BFR exercise (Peyrard et al., 2019). Brandner, Warmington et al. (2015) reported elbow flexion resistance exercise with BFR (20% 1‐repetition maximum) to significantly increase corticospinal excitability for ≥60 min, to a greater extent than unrestricted heavy and low external‐load training. Notably, continuous BFR induced significantly larger and longer increases in corticospinal excitability (≥60 min vs. 40 min) compared to higher pressure intermittent BFR (130% systolic blood pressure: 153 ± 5 mmHg; 8 cm bladder width), where cuffs are deflated during rest periods. This suggests continuous BFR, even at lower pressures (80% systolic blood pressure: 94 ± 4 mmHg; 8 cm bladder width), may better enhance group III/IV afferent firing, thereby augmenting corticospinal excitability. Currently, further comparison of the neuroplastic responses between different BFR parameters (e.g., applied pressure) remains limited (Table 1). One study does imply that lower prescribed BFR pressures (40% vs. 80% LOP) may reduce inhibition to a greater extent than higher prescribed BFR pressures, though the lack of a statistically significant interaction effect does not definitively infer a greater efficacy of lower cuff pressure (Norbury et al., 2024). When continuous BFR was applied at 70% AOP during 15 min of aerobic arm cycling (30% maximum power output), acute increases in corticospinal excitability were observed for ≥15 min following exercise, which was longer than unrestricted exercise that reduced to baseline levels 15 min post (Frechette et al., 2025). Conversely, Peyrard et al. (2019) reported a lack of change in MEP amplitude or inhibition following repeated arm cycling sprints to exhaustion with BFR. The absence of change in corticospinal excitability could be attributed to the exercise protocol (i.e., fatiguing exercise) or the singular time point at which corticospinal excitability was measured (2 min following exhaustion). Consequently, increases in corticospinal excitability may have been transiently attenuated by peripheral fatigue with reported reductions in maximal M‐wave response via peripheral nerve stimulation (Peyrard et al., 2019).

**TABLE 1:** Studies assessing the acute neurophysiological responses to blood flow restriction exercise.

Study	Participant information	Exercise type	Exercise condition	Blood flow restriction parameters	Neurophysiological assessments	Findings
Brandner, Warmington et al. (2015)	*n*: 10 males *Status*: unresistance trained *Age*: 22 ± 2 years	Unilateral elbow flexion/ extension	*Heavy‐load*: 4 × 6–8 repetitions at 80% 1RM, 2.5 min inter‐set rest *Low‐load*: 1 × 30, 3 × 15 repetitions, at 20% 1RM, 30 s inter‐set rest *Low‐load BFR‐continuous (BFR‐C)*: 1 × 30, 3 × 15 repetitions at 20% 1RM, 30 s inter‐set rests *Low‐load BFR‐intermittent (BFR‐I)*: 1 × 30, 3 × 15 repetitions at 20% 1RM, 30 s inter‐set rests	Pneumatic tourniquet cuff: 52 cm long, 10.5 cm wide; bladder length 45 cm, bladder width 8 cm *BFR‐C*: cuff remains inflated throughout the entire exercising protocol (80% systolic blood pressure: 94 ± 4 mmHg) *BFR‐I*: cuff is deflated during rest periods (130% systolic pressure: 153 ± 5 mmHg)	*Measure*: MEP *Trials*: 10 *State*: active (3.93 ± 0.4% EMG) *Test stimulus*: 130% AMT	**Within‐group comparisons** ↔ Heavy‐load ↑ Low‐load (at 5 and 20 min)^***^ ↑ BFR‐C (at 5, 20, 40 and ≥60 min)^***^ ↑ BFR‐I (at 5, 20 and 40 min)^***^ **Between‐group comparisons** Low‐load > heavy‐load (5, 20 and 40 min)^*^ BFR‐C > heavy‐load (5, 20, 40 and 60 min)^***^ BFR‐C > low‐load (5^**^, 20^***^, 40 and 60 min^**^) BFR‐C > BFR‐I (5^*^, 20^*^, 40^***^ and 60 min^***^)
					*Measure*: SICI *Trials*: 10 *State*: active (3.93 ± 0.4% EMG) *Test stimulus*: 130% AMT *Conditioned stimulus*: 70% AMT *Interstimulus interval*: 3 ms	**Within and between group comparisons** ↔ All exercise conditions
					*Measure*: *M*_max_ *Trials*: 5 *State*: rested *Test stimulus*: 120% maximal response	**Within and between‐group comparisons** ↔ All exercise conditions
Frechette et al. (2025)	*n*: 12 males *Status*: recreationally active and novice to arm cycling *Age*: 24 ± 4 years	Arm cycling	*High intensity*: 15 min at 60% maximum power at ${\dot{V}}_{O_{2}\mathrm{peak}}$ *Low intensity*: 15 min at 30% maximum power at ${\dot{V}}_{O_{2}\mathrm{peak}}$ *Low intensity BFR*: 5 min at 30% maximum power at ${\dot{V}}_{O_{2}\mathrm{peak}}$ *BFR‐only*: no exercise; 15 min sat with arms on ergometer	Pneumatic tourniquet cuff: 83 cm long, 5 cm wide BFR applied continuously at 70% AOP	*Measure*: MEP *Trials*: 25 *State*: active (5% MVC) *Test stimulus*: 130% AMT	**Within‐group comparisons** ↔ High intensity (at 1 min) ↑ High intensity (at 10 and ≥15 min)^***^ ↔ Low intensity (at 1 and 15 min) ↑ Low intensity (at 10 min)^**^ ↔ Low intensity BFR (at 1 min) ↑ Low intensity BFR (at 10 and ≥15 min)^***^ ↔ BFR‐only (at all time points)
					*Measure*: cSP *Trials*: 25 *State*: active (5% MVC) *Test stimulus*: 130% AMT	**Within‐group comparisons** ↔ High intensity (at 1 min) ↓ High intensity (at 10 ^***^ and ≥15 min^**^) ↔ Low intensity (at 1 and ≥15 min) ↓ Low intensity (at 10 min)^***^ ↔ Low intensity BFR (at 1 and 10 min) ↑ Low intensity BFR (at ≥15 min)^***^ ↔ BFR‐only (at all time points)
					Association (MEP and cSP)	No associations between MEP and cSP data
Norbury et al. (2024)	*n*: 12 males *Status*: recreationally active *Age*: 29 ± 6 years	Unilateral leg press	*Heavy‐load*: 4 × 10 repetitions at 70% 1RM, 53 s inter‐set rest *Low‐load*: 1 × 30, 3 × 15 repetitions at 30% 1RM, 30 s inter‐set rest *Low‐load BFR40%*: 1 × 30, 3 × 15 repetitions at 30% 1RM, 30 s inter‐set rest *Low‐load BFR80%*: 1 × 30, 3 × 15 repetitions at 30% 1RM, 30 s inter‐set rest	Pneumatic, contour tourniquet cuff: 11.5 cm width, 86 cm length BFR applied continuously at 40% and 80% LOP	*Measure*: MEP *Trials*: 10 *State*: active (10% MVC) *Test stimulus*: 130% and 150% AMT	**Within and between‐group comparisons** ↔ All conditions (at 130% and 150% AMT)
					*Measure*: cSP *Trials*: 10 *State*: active (10% MVC) *Test stimulus*: 130% and 150% AMT	**Within‐group comparisons** ↔ All conditions (at 130% and 150% AMT) **Between‐group comparisons** ↓ cSP (at 130% AMT) was greater for low‐load^**^, low‐load BFR40%^***^ and BFR80%^**^ vs. heavy‐load ↓ cSP (at 150% AMT) was greater for low‐load BFR40% vs. heavy‐load^**^ and low‐load BFR80%^*^
					*Measure*: SICI *Trials*: 10 *State*: active (10% MVC) *Test stimulus*: 130% AMT *Conditioned stimulus*: 70% AMT *Interstimulus interval*: 3 ms	**Within and between‐group comparisons** ↔ All conditions
					*Measure*: *M*_max_ *Trials*: 5 *State*: rested *Test stimulus*: 120% maximal response	**Within and between‐group comparisons** ↔ All conditions
					*Measure*: VA *Trials*: 1 *State*: active (100% MVC) *Test stimulus*: 120% maximal response	**Within and between‐group comparisons** ↔ All conditions
Peyrard et al. (2019)	*n*: 14 (Females = 4) *Status*: recreationally active *Age*: 26 ± 4 years	Arm cycling (repeated 10 s sprints)	*Normoxia*: repeated sprints until task failure (volitional stop or unable to maintain >70 rpm, 20 s active recovery) *Normoxia with BFR*: repeated sprints until task failure (volitional stop or unable to maintain >70 rpm, 20 s active recovery) *Hypoxia*: repeated sprints until task failure (volitional stop or unable to maintain >70 rpm, 20 s active recovery) *Hypoxia with BFR*: repeated sprints until task failure (volitional stop or unable to maintain >70 rpm, 20 s active recovery)	Pneumatic tourniquet cuff: 3 cm wide BFR applied continuously at 45% AOP (95.4 ± 11.9 mmHg)	*Measure*: MEP *Trials*: 10 *State*: active (10% MVC) *Test stimulus*: 140% AMT	Not reported
					*Measure*: MEP *Trials*: 2 per MVC intensity *State*: active (50, 75 and 100% MVC) *Test stimulus*: 140% AMT	**Between‐group comparisons** ↔ in change between BFR conditions ↑in MEP was larger for normoxia without BFR vs. Hypoxia (at 50%^*^ and 75%^*^ MVC, but not 100% MVC)
					*Measure*: cSP *Trials*: 10 *State*: active (10% MVC) *Test stimulus*: 140% AMT	**Between‐group comparisons** ↔ in change between conditions
					*Measure*: SICI *Trials*: 10 *State*: active (10% MVC) *Test stimulus*: 140% AMT *Conditioned stimulus*: 70% AMT *Interstimulus interval*: 3 ms	**Between‐group comparisons** ↔ in change between conditions
					*Measure*: *M*_max_ *Trials*: 2 *State*: rested *Test stimulus*: 130% maximal response	**Between‐group comparisons** ↓ in *M*_max_ was larger for BFR conditions vs. without BFR^**^
					*Measure*: VA_TMS_ *Trials*: 1 *State*: active (50, 75 and 100% MVC) *Test stimulus*: 140% AMT	**Between‐group comparisons** ↔ in change between conditions

^*^ *P* ≤ 0.05; ^**^ *P* ≤ 0.01; ^***^ *P* ≤ 0.001. ↑ = increase; ↔ = unchanged/no difference; ↓ = decrease. Abbreviations: 1RM, one‐repetition maximum; AMT, active motor threshold; AOP, arterial occlusion pressure; BFR, blood flow restriction; cSP, cortical silent period; EMG, electromyography; LOP, limb occlusion pressure; MEP, motor evoked potential; *M*_max_, maximal muscle response; MVC, maximal voluntary contraction; *n*: number of participants; SICI, short‐interval intracortical inhibition; VA, voluntary activation; VA_TMS_, voluntary activation via transcranial magnetic stimulation; ${\dot{V}}_{O_{2}\mathrm{peak}}$, peak volume of oxygen.

Following chronic heavy external‐load resistance training, increased corticospinal excitability has been accompanied by reduced short‐interval intracortical inhibition (SICI) and reduced cortical silent period (cSP) duration (Siddique et al., 2020). Interestingly, acute responses to heavy external‐load resistance training typically report a reduction in cSP duration, with no change in SICI (Latella et al., 2017), suggesting that plasticity responses to SICI occur later. Following acute BFR leg press (30% 1‐repetition maximum), cSP duration was reduced more than high external‐load leg press, but SICI was unchanged in BFR and heavy external‐load conditions (Norbury et al., 2024), consistent with earlier findings (Brandner, Warmington et al., 2015). The different responses in cortical inhibition may be mediated via the different mechanisms between SICI and cSP, as SICI is mediated by γ‐aminobutyric acid‐A (GABA_A_) receptors and the latter component of the cSP is mediated by GABA_A_ and GABA_B_ receptors (Konstantinović et al., 2023). Nevertheless, Frechette et al. (2025) reported an increase in cSP duration, despite also reporting increases in corticospinal excitability. Taken together, it is possible that a reduction in cSP, or other inhibitory circuits (e.g., long‐interval intracortical inhibition), or an increase in intracortical facilitation, underpins the increased corticospinal excitability following different types of BFR exercise. Additionally, BFR exercise may induce changes spinally, considering group III/IV afferents impact spinal excitability (Copithorne et al., 2020; Martin et al., 2006), and heightened spinal excitability is reported following acute (Mason et al., 2019) and chronic (Gomez‐Guerrero et al., 2024) heavy external‐load resistance exercise.

## 4 FUNCTIONAL ADAPTATIONS

Neuroplastic adaptations contribute to improved motor function (Dimyan & Cohen, 2011). Given the evidence reported above, BFR exercise may induce functionally beneficial neuroplasticity via long‐term potentiation. Improved functional ability is well documented following BFR exercise, with comparable improvements to high external‐load/intensity exercise in healthy (Laurentino et al., 2012) and clinical populations (Ahmed et al., 2024). In stroke patients, 5 weeks of low external‐load BFR resistance training improved gait speed, stride length, cadence, dynamic balance and strength comparable to heavy external‐load training (40% vs. 80% 1‐repetiton maximum) (Ahmed et al., 2024). Functional independence, measured via the Barthel index, also improved, suggesting improved quality of life. Similar improvements are reported in multiple sclerosis patients: 6 weeks of BFR walking (twice weekly; 15 × 1 min intervals) with progressive cadence increases (from 60 steps/min) improved gait speed, 6‐min walk distance, and sit‐to‐stand performance relative to unrestricted walking (40 min at ∼8/10 rating of perceived exertion) (Lamberti et al., 2020). A systematic review further supports BFR as a safe technique to improve sensorimotor function and quality of life, with no adverse events reported across seven studies within neurological disorder populations (Vinolo‐Gil et al., 2022).

In populations recovering from musculoskeletal injury, BFR exercise is an effective rehabilitation intervention (Hughes et al., 2019). Following anterior cruciate ligament reconstruction, BFR resistance training increased strength and hypertrophy comparable to traditional resistance training, with greater improvements in physical function and reduced knee pain (Hughes et al., 2019). Unfortunately, evidence for BFR exercise following spinal cord injury, where corticospinal reorganisation is critical for recovery, remains limited. Nonetheless, within motor‐impaired, spinal cord injury patients, leg extension and curl exercise at 30% 1‐repetition maximum with BFR (40% AOP) significantly increased muscle thickness and improved 6 min walking distance (50.3 m), 10 m walking time (−1.67 s), and timed up and go performance (−1.86 s) (Jønsson et al., 2024). Although no between‐group differences were observed between the BFR and unrestricted conditions, only the BFR group showed significant within‐group improvements on all gait performance metrics. In the upper limbs, greater strength gains were observed following forearm extensor and flexor exercise at 30% 1‐repetition maximum with BFR (60% LOP) compared to unrestricted exercise at 50% 1‐repetition maximum (Shadgan et al., 2024). These findings highlight that BFR enables participants to use lower external loads while achieving functional adaptations in those with motor‐impaired injuries.

Given the benefits outlined above, BFR exercise may also promote healthy ageing. Ageing is associated with decreases in muscle mass, strength and motor control, partly due to reduced corticospinal excitability and afferent feedback (Baudry et al., 2015; Clark & Taylor, 2011). Age‐related reductions in Ia afferent feedback have been reported in humans (Baudry et al., 2015), with animal models indicating similar declines in III/IV afferent feedback (Caron et al., 2018). Therefore, BFR exercise may provide sufficient group III/IV afferent stimulation to induce neuroplasticity in older adults. Muscular adaptations following BFR exercise in older adults are better understood. A meta‐analysis indicated that BFR resistance training in adults aged 50–86 years induces greater strength increases than unrestricted, low external‐load matched exercise, with comparable muscular hypertrophy to high external‐load resistance training (Centner et al., 2019). Furthermore, BFR walking resulted in greater increases in strength and muscle mass than unrestricted walking. Considering its accessibility, these data highlight that BFR walking can enhance muscular adaptations in those who are contraindicated to high external‐load/intensity exercise or low external‐load resistance training.

**FIGURE 1:**
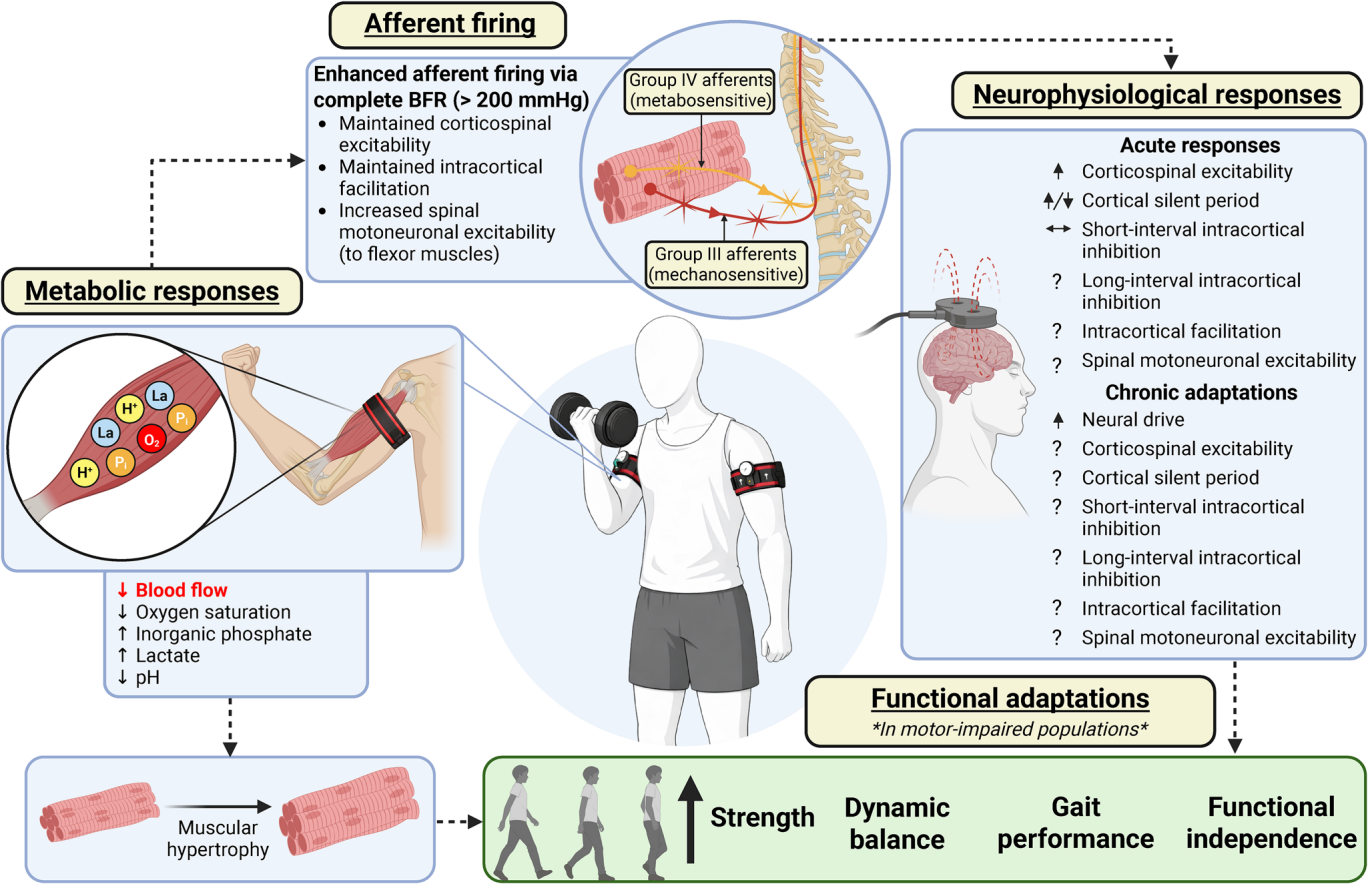
Translational perspectives on neurophysiological responses and mechanisms in blood flow restriction exercise. Reduced blood flow induces metabolic perturbations that promote muscular hypertrophy and enhance group III/IV afferent firing, leading to neurophysiological responses that may improve functional ability. Dotted arrows indicate proposed links; ↑ = increase; ↔ = unchanged; ↓ = decrease; ? = untested. Created in BioRender (https://BioRender.com/4ux33it).

Collectively, aerobic or resistance exercise with BFR appears to improve muscular strength, hypertrophy and functional ability in patients with neurological disorders, motor‐impaired injuries and older adults, comparable to high external‐load/intensity exercise (Figure 1). However, further research is warranted, particularly in neurological disorders and spinal cord injury populations, to determine the extent to which BFR exercise can enhance functional performance and the underpinning mechanisms.

## 5 FUTURE DIRECTIONS

Our knowledge of muscular adaptations to BFR exercise is developing, with increasing evidence highlighting its efficacy to improve functional abilities in those who are contraindicated to high external loads/intensities. However, the neurophysiological adaptations remain underexplored. Studies investigating maintained afferent firing via BFR used non‐individualised, supra‐occlusive pressures (>200 mmHg) (Copithorne et al., 2020; Latella et al., 2020, 2021; Martin et al., 2006), exceeding pressures recommended by current BFR guidelines (Patterson et al., 2019). Therefore, future research should apply clinically relevant pressures (40–80% LOP/AOP) to determine their ability to enhance afferent firing. Additionally, current insights into BFR exercise‐induced neuroplasticity derive from acute investigations using limited neurophysiological measures. Given the balance between inhibitory and facilitatory circuits and the excitability of subcortical regions influence exercise‐induced neuroplasticity, future research should incorporate comprehensive testing measures that assess inhibitory and facilitatory circuits, both cortically and subcortically, using a range of TMS measures and electrical spinal stimulations. Finally, although BFR exercise has been advocated as safe and feasible in clinically injured and neurologically impaired populations (Vinolo‐Gil et al., 2022), supporting evidence remains scarce. Safety concerns have been raised over potential adverse cardiovascular events mediated by the autonomic nervous system, particularly via activation of the exercise pressor reflex resulting from group III and IV afferent firing during BFR exercise (Spranger et al., 2015). Adding BFR to low external‐load exercise is reported to exacerbate heart rate, blood pressure and cardiac output compared to external‐load matched exercise; however, when moderate pressures are applied (∼60% LOP), these autonomic responses are modest compared to heavy‐load exercise in healthy individuals (Brandner, Kidgell et al., 2015). Future research is required to understand the risk of adverse events when BFR exercise is employed in those susceptible to exacerbation of pre‐existing symptoms.

## 6 CONCLUSION

Exercising with BFR induces substantial muscular adaptations and improved functional ability in individuals who may not tolerate high external‐load/intensity exercise. Emerging evidence suggests that BFR exercise may induce functionally beneficial neuroplasticity, with significant implications for those with motor impairment. These neurophysiological responses are thought to result from enhanced group III afferent firing during contractions and enhanced group IV afferent firing driven by sustained metabolic stimuli during BFR application. This short commentary highlights BFR exercise as a potentially novel approach to overcome barriers to exercise‐related benefits in motor‐impaired populations. However, critical gaps in understanding the neurophysiological responses and adaptations remain. Future work should conduct comprehensive assessments of neurophysiological responses, prioritising chronic adaptations, investigate whether recommended BFR pressures (40–80% LOP/AOP) enhance afferent firing, and further evaluate the safety and efficacy of BFR exercise in motor‐impaired populations.

## AUTHOR CONTRIBUTIONS

All the authors included within this study (Kai T. Fox, Luke Hughes, Brendan R. Scott, Paul S. R. Goods, Shaun Y. M. Teo and Ann‐Maree Vallence) contributed to the conception or design of the work, the acquisition, analysis or interpretation of data for the work, and drafting the work or revising it critically for important intellectual content. All the authors have read and approved the final version of the manuscript and agree to be accountable for all aspects of the work in ensuring that questions related to the accuracy or integrity of any part of the work are appropriately investigated and resolved, and all persons designated as authors qualify for authorship, and all those who qualify for authorship are listed.

## CONFLICT OF INTEREST

None declared.
